# Fighting HIV-1 Persistence: At the Crossroads of “Shoc-K and B-Lock”

**DOI:** 10.3390/pathogens10111517

**Published:** 2021-11-20

**Authors:** Chiara Acchioni, Enrico Palermo, Silvia Sandini, Marta Acchioni, John Hiscott, Marco Sgarbanti

**Affiliations:** 1 Department of Infectious Diseases, Istituto Superiore di Sanità, Viale Regina Elena 299, 00161 Rome, Italy; chiara.acchioni@iss.it (C.A.); silvia.sandini@iss.it (S.S.); marta.acchioni@iss.it (M.A.); 2 Istituto Pasteur Italia—Cenci Bolognetti Foundation, Viale Regina Elena 291, 00161 Rome, Italy; enrico.palermo@istitutopasteur.it (E.P.); john.hiscott@istitutopasteur.it (J.H.)

**Keywords:** HIV-1 latency, CD4^+^ T cell reservoirs, LRAs, LPAs, Shock and Kill, Block and Lock, immunostimulatory agents, LTR enhancer, NF-κB, p50, c-Jun, Tat

## Abstract

Despite the success of highly active antiretroviral therapy (HAART), integrated HIV-1 proviral DNA cannot be eradicated from an infected individual. HAART is not able to eliminate latently infected cells that remain invisible to the immune system. Viral sanctuaries in specific tissues and immune-privileged sites may cause residual viral replication that contributes to HIV-1 persistence. The “Shock or Kick, and Kill” approach uses latency reversing agents (LRAs) in the presence of HAART, followed by cell-killing due to viral cytopathic effects and immune-mediated clearance. Different LRAs may be required for the in vivo reactivation of HIV-1 in different CD4^+^ T cell reservoirs, leading to the activation of cellular transcription factors acting on the integrated proviral HIV-1 LTR. An important requirement for LRA drugs is the reactivation of viral transcription and replication without causing a generalized immune activation. Toll-like receptors, RIG-I like receptors, and STING agonists have emerged recently as a new class of LRAs that augment selective apoptosis in reactivated T lymphocytes. The challenge is to extend in vitro observations to HIV-1 positive patients. Further studies are also needed to overcome the mechanisms that protect latently infected cells from reactivation and/or elimination by the immune system. The Block and Lock alternative strategy aims at using latency promoting/inducing agents (LPAs/LIAs) to block the ability of latent proviruses to reactivate transcription in order to achieve a long term lock down of potential residual virus replication. The Shock and Kill and the Block and Lock approaches may not be only alternative to each other, but, if combined together (one after the other), or given all at once [namely “Shoc-K(kill) and B(block)-Lock”], they may represent a better approach to a functional cure.

## 1. Introduction

Infection with the human immunodeficiency virus (HIV-1), the lentivirus responsible for Acquired Immunodeficiency Syndrome (AIDS), appeared 40 years ago and continues to be a never-ending global threat for the world population. The death toll has reached an estimate of 34.7 million since the start of the pandemic, with approximately 37.6 million people living with HIV in 2020 (https://www.unaids.org/en/resources/fact-sheet, accessed on 6 October 2021). Since the mid-1990s, Highly Active Antiretroviral Therapy (HAART), a combination of antiretroviral drugs targeting different steps of the HIV-1 life cycle, has been the standard of care for patients living with HIV-1. More than 30 antiretroviral drugs have been approved by the Food and Drug Administration (FDA), including non-nucleoside reverse transcriptase inhibitors, nucleoside reverse transcriptase inhibitors, integrase inhibitors, protease inhibitors, and virus entry inhibitors (https://hivinfo.nih.gov/understanding-hiv/fact-sheets/fda-approved-hiv-medicines, accessed on 6 October 2021). While the original HAART regime was difficult to adhere to because of the complexities associated with multiple pills per day, currently, a single-tablet regimen (STRs) [1] or long-acting, injectable treatments have become available [2]. Moreover, by the end of 2020, an estimated number 27.4 million people had access to antiretroviral therapy (https://www.unaids.org/en/resources/fact-sheet, accessed on 6 October 2021).

HAART effectively decreased the viral load in HIV-1 infected patients to undetectable levels, and contributes to a partial reconstitution of the immune system, as demonstrated by an increase in the number and function of circulating CD4^+^ T cells [3,4]. Therefore, HAART blocks or slows the progression of AIDS, and HIV transmission, especially when started at higher CD4^+^ T cell counts [5]. Moreover, HAART proved effective in preventing HIV-1 infection among discordant couples [6,7], while Pre-Exposure Prophylaxis (PrEp), consisting of a combination of two drugs, provides a variable degree of protection in people at a high risk of HIV-1 infection [8].

Despite the undisputed achievement of HAART, HIV-1 patients undergoing HAART treatment experience non-AIDS-related comorbidities (NARC), especially above the age of 50 years. This particular seropositive population tends to age more rapidly than non-HIV-1 infected individuals, with the earlier onset of morbidities typically associated with aging, like non-AIDS-related malignancies [9], arterial hypertension [10], cardiovascular diseases [11], diabetes mellitus [12], kidney diseases [13], dyslipidemia [14], liver-related diseases [15], and low bone mineral density [16]. 

HIV-1 infection itself, despite the suppression of the viral load by HAART, is responsible for specific morbidities like HIV-related pulmonary arterial hypertension (HRPAH), caused by HIV-mediated chronic inflammation, immune activation, and HIV-mediated pulmonary vascular remodeling [17]. This scenario is worsened in the presence of co-infections with either hepatitis C or B viruses (HCV/HBV), or with mycobacterium tuberculosis (Mtb) [18]. It is presently unclear if the global COVID-19 pandemic will contribute to worsening outcomes in people living with HIV (PLWH). In this regard, immune suppression in PLWH could increase susceptibility to the SARS-CoV-2 infection; while some studies suggest that HIV patients with low CD4^+^ T cell counts display less severe SARS-CoV-2 infection symptoms [19], other results indicate an increased risk of death, after adjusting for age, sex, ethnicity, and comorbidities [20]. Interestingly, randomized clinical trials have demonstrated that the administration of HAART drugs, such as the protease inhibitor lopinavir together with ritonavir (LPV/r), did not improve COVID-19 outcomes or symptoms compared to the standard of care [21].

It is now widely accepted that HAART is unable to eradicate an established HIV infection [22,23,24], and therapy interruption results in a rebound in viremia and systemic infection [25,26,27,28]. A rebound after treatment interruption is due to the existence of long-lived stable HIV-1 cell reservoirs, generated early during the primary infection [29]. In these reservoirs, the integrated HIV-1 provirus is largely transcriptionally silent and not targeted by HAART. Moreover, latently infected cells express very limited levels of viral proteins, thus remaining invisible to the host immune system. The fact that large numbers of latently infected cells harbor defective proviruses indicates that only a few cells actually contribute to HIV-1 persistence [30]. Different cell types can serve as an HIV-1 reservoir, including macrophages, dendritic cells (DC), and microglial cells [31,32], but the long-lived resting CD4^+^ T cells are the most abundant and relevant cell type responsible for maintaining HIV-1 latency [33,34,35,36,37,38,39,40,41,42].

In the absence of a protective vaccine [43] and the failure of intensified HAART to eradicate the infection [44,45,46], novel therapeutic approaches are still required to eliminate HIV-1 provirus. Two main strategies are currently being pursued in the search for an HIV cure: eradication of the latent virus reservoirs or “sterilizing cure” and HAART-free control of HIV-1 replication, or a “functional cure”, in which the restoration of effective immune functions reduces the reservoir size, HIV-induced immune activation, and inflammation [47,48]. The sterilizing cure approach has important examples in the cases of the “Berlin patient” and the “London patient”, where HIV-1 was undetectable after allogenic bone marrow transplantations from homozygous CCR5∆32 donors [49,50]. Nevertheless, such an approach is not applicable on a large scale due to the high risk of the procedure and the paucity of donors bearing the HIV-1 resistant homozygous CCR5∆32 mutation. Therefore, the engineering of patient hematopoietic stem cells (HSC) to obtain a CCR5 deletion, or other gene modifications able to render CD4^+^ T cells resistant to HIV-1 infection, followed by an autologous infusion/transplantation has been proposed [51,52].

Due to the fact that HAART is most efficient at reducing the reservoir size when initiated early after HIV infection [53], a functional cure approach may come from the initiation of therapy during the very early primary stage of acute HIV infection. Post-treatment controllers (PTCs) from the VISCONTI cohort, where 14 HIV patients following HAART discontinuation maintained long lasting control of viremia, showed the potential efficacy of the approach [54]. Another approach is represented by the depletion of discrete T cell subsets carrying the integrated HIV-1 DNA based on the use of drugs with pro-oxidant activities taking advantage of metabolic imbalances occurring in latently infected cells [55,56,57,58].

To date, the “Shock or Kick, and Kill” approach has been the most studied of the “functional cure” approaches, with both promising and disappointing results [59,60]. The approach consists of the use of pharmacological stimulators known as latency reversing agents (LRAs), with the capacity to transcriptionally reactivate the latent provirus (the shock/kick phase) in the presence of HAART (to block reinfection of susceptible cells by the reactivated virus), followed by cell-killing due to viral cytopathic effects and/or immune-mediated clearance [61,62,63]. The LRA-mediated reactivation from latency is dependent on the engagement of signal transduction pathways typically involved in innate as well adaptive immune responses, culminating with the activation of cellular transcription factors and co-activators, responsible for the initiation/elongation of HIV-1 transcription, acting at the level of the 5′ long terminal repeats (LTR) of the integrated provirus. Several classes of LRAs have been employed for this purpose, including epigenetic drugs such as histone deacetylase inhibitors (HDIs), as well as protein kinase C agonists, bromodomain and extra-terminal motif (BET) protein inhibitors (BETis), activators of the Akt pathway, STAT5 sumoylation inhibitors, SMAC mimetics, and immunomodulators [64]. The alternative approach, named “Block and Lock” [65,66], is based on the rationale of blocking the reactivation of HIV-1 transcription from latently infected cells through pharmacologic inhibitors, for example, targeting Tat [67,68,69]; the final goal is a stable lock of proviral DNA due to an increase in permanent epigenetic silencing overtime, resulting in a HIV functional cure [70]. These next sections of this review will describe the current shock/kick and kill approaches and related immune pathway stimulation proposed to promote HIV-1 release from latency and subsequent cell-killing, as well as the apparently opposed Block and Lock approach.

## 2. Molecular Mechanisms of HIV-1 Latency Reversal

### 2.1. Mechanism of HIV-1 DNA Latency

Many factors contribute to HIV-1 silencing [71,72,73,74]. Retrotranscribed double stranded HIV-1 DNA can be present in infected cells in the form of linear, autointegrated (generated by the so called suicidal integration), 1- or 2-LTR circles, or proviral integrated DNA, with linear HIV-1 DNA serving as a precursor for the other forms [75,76]. Unintegrated circles are subjected to pre-integration mechanisms of latency, including epigenetic silencing through the assembly of histones with heterochromatin signatures [77]. These episomal HIV-1 genomes are unable to be replicated and mostly persist in non-dividing, or slowly dividing cells in vivo, as for example, naïve CD4^+^ T-cells, resting memory CD4^+^ T cells, and macrophages [76]. Post-integration latency represents the most important form of persistent HIV-1 DNA, able to replicate even in proliferating cells. HIV-1 mostly integrates into intronic regions of actively transcribed genes [78]. It was demonstrated that the expression of HIV-1 depends on the insertion site, with active transcription related to cellular promoter and enhancer proximity, while latent proviruses were mostly detected in genomic positions far from cellular promoters and enhancers [79]. However, when cellular promoter/enhancers are too close to the 5′ LTR, steric hindrance may occur if proviral integration is in the same transcriptional orientation as the cellular gene, resulting in the displacement of transcription factors from the LTR [80]. If the HIV-1 provirus is in an opposite orientation compared to the cellular gene, RNA-pol II from the two promoters (cellular and viral) can collide, resulting in transcriptional shut off, of both, the cellular gene and the HIV-1 genome, or at least of the genes bearing the promoter possessing a weaker activity (promoter occlusion) [80]. Another mechanism that may promote latency is the sequestration of the HIV-1 enhancer by a proximal cellular promoter, therefore failing at serving as a crucial sequence for HIV-1 LTR transcription initiation (enhancer trapping) [80]. Despite this scenario, latency can be reversed, if non-defective genomes are integrated, simply by the cooperative action of the viral transactivator Tat and cellular transcription factors and co-factors, resulting in a strong binding to the LTR and subsequent resistance to the transcription started at the cellular gene promoters. Other factors contribute to HIV-1 silencing like 3′ LTR antisense transcripts, the shortage of available cellular cofactors, suboptimal amounts of the viral trans-activator Tat, the presence of microRNA inhibiting nuclear export of viral transcripts, and chromatin modifications at the viral promoter [71]. Indeed, the epigenetic silencing of the integrated provirus also participates in shutting off viral transcription, thus contributing to HIV-1 latency. DNA hypermethylation at two CpG islands positioned close to the 5′ and 3′ of the LTR enhancer region, hypoacetylation, and methylation of histones, assembled in nucleosomes surrounding the HIV-1 LTR, and crotonylation represent hallmarks of heterochromatin at the integration site [75]. In this context, the role of chromatin environment in maintaining viral latency is widely described [74,81,82]. Structurally, chromatin is organized in nucleosomes, consisting of a section of 146 base pairs of DNA wrapped around an octamer of histone proteins, formed by two molecules of each core histone (H2A, H2B, H3 and H4) [83], whose activity can be regulated by post-translational modification (PTMs) resulting in a chromatin state transition and altered accessibility of DNA to transcription factors [83]. Interestingly, two nucleosomes, nuc-0 and nuc-1, have been found to be positioned in the 5′ LTR of HIV-1 [81], indicating a role in the modulation of viral promoter activity.

The positioning of nucleosomes within the genome is crucial for the control of gene expression and is regulated by different mechanisms. First, the activity of ATP-dependent chromatin remodeling complexes, which possess an ATPase domain to hydrolyze ATP and exploit the energy to move or remove nucleosomes [84]. Second, PTMs affecting the histone N-terminal tails, mainly acetylation and methylation, alter the interaction histones-DNA or histones-histones generating a “histone-code”, which serves as a docking site for proteins with chromatin remodeling activity [83]. Another mechanism of latency regulation is represented by the DNA CpG methylation, which, in the context of HIV-1 LTR, led to contrasting observations, with some studies supporting the thesis of an association of this modification with viral latency [85,86,87] and some confuting it [88,89]. 

Histone acetylation and methylation are the most described PTMs involved in the maintenance of HIV-1 latency [90]; histone acetylation results in a more accessible chromatin conformation and is catalyzed by Histone Acetyl Transferases (HATs), whereas Histone Deacetylases (HDACs) remove the acetyl groups leading to transcriptional silencing [91]. The recruitment of different HATs, such as p300/CBP, PCAF (P300 CBP-associated factor), Tip60, and GCN5 to the 5′ LTR of HIV-1 is regulated by the presence of Tat or by distinct stimuli involving the NF-κB signaling [92]. On the other hand, the activity of HDACs at the viral promoter is regulated by other cellular factors, including LSF-1/YY1, NF-κB p50/p50 homodimer, and CBF-1 [92]. In this case, several transcription factors such as NF-κB, NFAT, and c-Jun are unable to initiate transcription at the 5′ LTR, promoting viral latency [73]. In addition to HDACs, the activity of Histone Methyltransferases (HMTs) contributes to determining the transcriptional fate of latent provirus: the HMTs SUV39H1, a subunit of the polycomb repressive complex 2 (PRC2), and EZH2, catalyze the formation of H3K9me3 and H3K27me3, respectively [93,94], and are associated with maintenance of HIV-1 latency. Furthermore, the HMT G9a is involved in this process by catalyzing dimethylation of H3K9 [95].

Considering the critical contribution of PTMs in the induction or preservation of HIV-1 latency, pharmacological interventions have been pursued with the aim to perturbate the latent viral state and induce latency reversal.

An interesting feature of 0.5% of PLWH that do not require HAART to keep viral replication under control (also defined as elite controllers) is the presence of a high percentage of intact proviral HIV-1 DNA, potentially able to reactivate transcription [96,97]. Nevertheless, elite controllers’ integrated proviral DNAs express ten times less viral RNAs compared to people under HAART because of a deep state of latency, possibly due to a selective pressure exerted over time by a powerful anti-HIV-1 cellular response eliminating cells harboring HIV-1 sequences more prone to transcriptional reactivation [96,97]. Such residual deep latency is related to integration in non-protein coding areas of the host genome, such as chromosomal centromeric regions or in Krüppel-associated box domain-containing zinc finger genes on chromosome 19, characterized by a strong repression of transcription, with one patient almost considered functionally cured despite at least one intact proviral sequence [96,97]. Finally, non-intact defective proviral sequences do not contribute to a viral rebound after HAART interruption, and can suggest that a sterilizing cure is possible when they are the only traces left of an HIV-1 infection [97].

### 2.2. Cell Types Supporting HIV-1 Latency

CD4^+^ T lymphocytes represent the main target of HIV-1 latency [33,39,98]. Naïve T cells (T_N_), central memory T cells (T_CM_), transitional memory T cells (T_TM_), effector memory T cells (T_EM_), and stem cell like memory T cells (T_SCM_), all represent distinct stages of differentiation that are capable of becoming latently infected HIV reservoirs possessing different relevance as reservoirs due to their respective different half-life and their self-renewal ability [71]. Latently infected CD4^+^ T cells can also be found among other functional subsets, including regulatory T cells (Treg), Th17 memory cells, T follicular helper cells (T_fh_), and resident memory CD4^+^ T cells (T_RM_) [71].

Other sources of viral rebound upon HAART treatment interruption are represented by non-T cell reservoirs like macrophages, dendritic cells (DCs) and microglial cells, while follicular dendritic cells (fDCs) can trap and transfer HIV-1 to CD4^+^ T cells in secondary lymphoid organs [71].

HIV-1 latency reduces to a minimum HIV-1 transcription initiation and elongation, post-transcriptional processing, and translation of viral proteins. LRA-mediated HIV-1 escape from latency involves the activation of immune-related signaling pathways, particularly those involving the interaction between CD4^+^ T cells and antigen presenting cells (APC). An important consideration for LRA drugs is reactivation of viral transcription and replication without causing a generalized immune activation [99] and life threatening toxicity, as is seen with anti-CD3 antibodies [100].

### 2.3. Cellular Transcription Factors Mediating Viral Transcription Initiation in HIV-1 Latently Infected CD4^+^ T Cells

An initial HIV-1 transcription depends upon cellular transcription factors, before the viral transactivator protein Tat accumulates [80]. Once Tat becomes available, it binds the CycT1 subunit of the P-TEFb elongation complex, also formed by the cyclin-dependent kinase (CDK9), together with the transactivation-responsive element (TAR) at the 5′ end of HIV transcripts, dramatically increasing the rate of transcription [101,102]. The HIV-1 LTR promoter region contains several cellular transcription factor binding sites: (i) nuclear factor of activated T cells (NFAT); (ii) the activator protein-1 (AP1), composed of c-Jun homodimers or c-Jun/c-Fos heterodimers; (iii) the nuclear factor κB kappa-light-chain-enhancer of activated B cells (NF-κB), a family of transcription factors able to dimerize in different combinations; (iv) the interferon regulatory factors (IRFs); (v) the signal transducer and activator of transcription (STAT)-5; and several others [80]. Despite this plethora of potential sites and factors, only the enhancer binding NF-κB sites are essential for the inducible reactivation in a primary CD4^+^ T central memory (T_CM_) cell latency model [103].

The limited availability of these transcriptional regulators may determine a strong reduction in viral transcription, as in the case of viral shut-off during the transition from activated to resting memory T cells [104]. In these cells, cellular factors can be hijacked in the cytosol, synthesized and/or activated at suboptimal amounts, or sequestered into inactive protein complexes. This cellular localization of NF-κB family members, particularly the RelA transcription activator, is restricted to the cytoplasm by the binding to the IκB inhibitory proteins. Moreover, in HIV-1-infected resting cells, the inhibitory p50/p50 homodimer, lacking the transactivation domain present in the p50/RelA NF-κB complex, binds the enhancer sequence repressing transcription, additionally due to the recruitment of HDAC1 to the 5′ LTR [105]. Interferon regulatory factor 8 (IRF-8), described by our group in Jurkat [106], and by others in U1 cells [107] as an inhibitor of LTR-mediated transcription, has been recently suggested to induce HIV-1 latency in macrophages following the interactions with commensal and pathogenic bacteria [108]. Other negative regulators of HIV-1 transcription were also recently identified by using -omics and functional approaches, suggesting a role for them in latency maintenance. Such factors include the Krüppel-like C2H2 zinc finger DNA-binding proteins KLF2 and KLF3, that recognize GC-rich regions conserved in HIV-1 and HIV-2 LTRs; KLF2 and KLF3 are both able to repress HIV-1 and HIV-2 transcription in CD4^+^ T cells [109]. A whole-genome CRISPR knockout screen in infected T cells identified the zinc-finger protein 304 (ZNF304) as a binder of the HIV LTR around the enhancer NF-κB sites, and also acted as a promoter of latency by recruiting histone methyltransferases in association with TRIM28 [110]. Unrecognized host factors promoting HIV-1 latency, like FTSJ3, TMEM178A, NICN1, and the Integrator Complex, have been also identified by using the “Reiterative Enrichment and Authentication of CRISPRi Targets” (REACT) novel screening strategy [111].

CD4^+^ T cell activation triggers the phosphorylation and the subsequent ubiquitination and degradation of IκB-α, resulting in the nuclear accumulation of the heterodimer p50/RelA and the subsequent displacement of the p50/p50 homodimer from the enhancer sequence. The recruitment of histone acetyl transferases (HATs) remodels Nuc1 to a conformation that allows viral transcription to proceed [80]. Moreover, the acetylation of RelA by p300/CBP HATs at lysine 218, 221, and 310 affects NF-κB transcriptional activity; the acetylation of lysine 221 enhances DNA binding and impairs interaction with IκB-α, while the acetylation of lysine 310 promotes full NF-κB transcriptional activity [112,113].

The Ca^2+^/Calcineurin (CaN)-dependent transcription factor NFAT also binds the κB sites present in the enhancer region [114,115], recruits HATs [116], and promotes viral transcription [117]. Nevertheless, the role of NFAT in the reactivation of HIV-1 replication from latency with activators of the Ca^2+^ pathway and PKC activators has been questioned [118]. Other transcription factors, like AP1 and IRF-1, have been shown to bind the LTR enhancer in association with activated NF-κB, further promoting HIV-1 transcription [119,120]. The diverse cellular environment, corresponding to distinct differentiation stages of CD4^+^ T cells, seems to determine the relative importance of each of these factors in promoting HIV-1 escape from latency [121]. For example, the use of specific inhibitors in the NF-κB pathway prevents the blocking of HIV-1 reactivation in an HIV-1 latency model resembling T_CM_ cells, stimulated by TCR engagement or phytohemagglutinin (PHA) [103]. 

Another important factor driving HIV-1 into the latent, transcriptionally silent state is the availability of the Tat protein; low transcription initiation rates or mutated Tat protein can also contribute to the emergence of the latent state [122]. The resting state of CD4^+^ T cells is crucial for HIV-1 to enter into latency, and the activation of these cells is essential to promote latency escape. Nevertheless, stochastic fluctuations of the Tat protein appear to be sufficient to determine the fate of viral transcription independently from the cellular activation state [123]. Furthermore, RNA polymerase II pausing, another process potentially impacting HIV-1 latency reversal, appears to be a stochastic phenomenon, with only a small percentage of transcripts pausing even in the absence of Tat, therefore further sustaining the hypothesis of randomly occurring events regulating HIV-1 transition from latency to active replication [124].

### 2.4. Elongation of Viral Transcripts during HIV-1 Latency Reversal in CD4^+^ T Cells

RNA Pol II is initially paused at position +30 to +60 from the initial transcriptional start site, but is able to proceed to position +60–+90 [125], where it pauses again, possibly due to a secondary structure, the pause hairpin, that forms alternatively to the TAR RNA element [126], and to the elongation repressing activity of the negative elongation factor (NELF) [127] and the DRB sensitivity inducing factor (DSIF) [128], even if the role of NELF in RNA pol II pausing is questioned [125]. The efficient elongation of viral transcripts requires the transcription elongation factor b (P-TEFb), composed of cyclin T1 and CDK9 [129,130,131]. In resting CD4^+^ T cells, Cyclin T1 and activated CDK9 are expressed at low levels, and accumulate upon T cell receptor engagement [132]. P-TEFb is sequestered in a ribonucleoprotein complex (RNP) comprising 7SK RNA and 7SK binding proteins as EXIM-1 or HEXIM-2, 7SK methylphosphate capping enzyme (MePCE), and La ribonucleoprotein domain family, member-7 (LARP7) [133,134,135,136]. In the absence of Tat, efficient PTEF-b recruitment and the LTR transcriptional elongation of a latent provirus are detected after TCR-mediated activation, with the release of PTEF-b from the inhibitory complex containing 7SK RNPs [137]. The released P-TEFb is recruited by Bromodomain-containing protein 4 (BRD4) to acetylated histones and also to Lys 310 acetylated RelA, to promote transcription elongation, particularly of NF-κB regulated genes [121].

**Figure 1:**
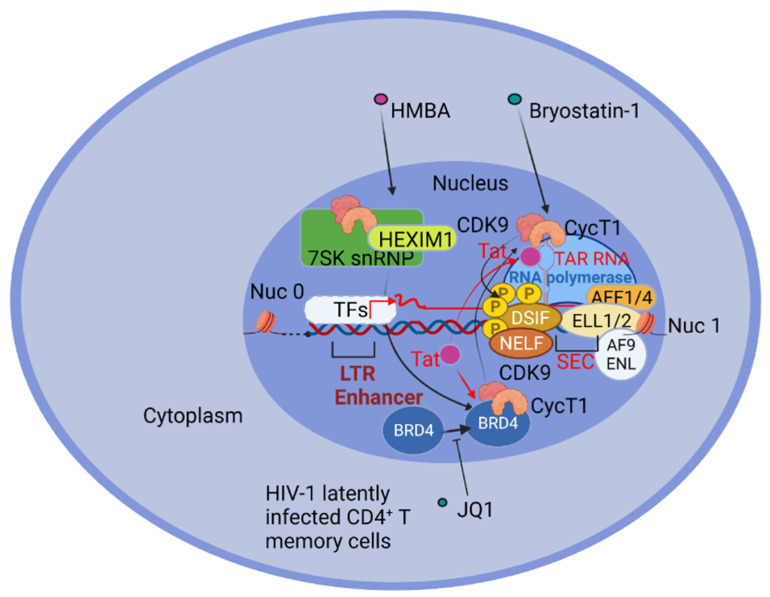
Schematic representation of the HIV-1 transcription elongation process in a generic CD4^+^T memory cell, latently infected. Upon transcription initiation mediated by transcription factors (TFs) binding the Long Terminal Repeats (LTR) enhancer, a proportion of the viral transcripts paused forming the TAR RNA structure. The elongation factor PTEF-b, made by CycT1 and CDK9, is hijacked in the 7SK snRNP/HEXIM1 inhibitory complex in resting CD4^+^ T memory cells. The treatment of these cells with Hexamethylenebisacetamide (HMBA) determines PTEF-b release from the inhibitory complex. PTEF-b can be bound to the Bromodomain-containing protein (BRD) 4, thus stimulating transcription elongation from cellular genes. As soon as the viral transactivator Tat accumulates, it displaces PTEF-b from BRD4 to delocalize it to the HIV-1 Trans-activation response element (TAR), thus strongly promoting HIV-1 transcription elongation and virus replication. Bryostatin-1 treatment increases the level of available CycT1 and CDK9 in resting memory CD4^+^ T cells, while the BRD4 inhibitor JQ1 prevents BRD4 from binding PTEF-b. Also indicated are the AFF1/4, ELL1/2, and AF9/ENL factors forming, together with PTEF-b and Tat, the multi-subunit complex named the “super elongation complex” (SEC). The negative elongation factor (NELF), and the 5,6-dichloro-1-β-D-ribofuranosylbenzimidazole (DRB) sensitivity inducing factor (DSIF), are phosphorylated by CDK9 determining NELF release and DSIF turned into a positive elongating factor. CDK9 also phosphorylates the largest subunit of RNA polymerase II (Pol II), thus allowing transcription elongation to proceed.

Once available, Tat competes with BRD4 for P-TEFb binding, as demonstrated by using the BRD4 inhibitor JQ1 that displaces BRD4 from the LTR region of HIV-1 chromatin and increases the association of P-TEFb with Tat [138]. The Tat displacement of BRD4 from P-TEFb acts to hijack P-TEFb away from cellular promoters [139,140]. In the presence of Tat, P-TEFb interacts with AFF1/4, ELL1/2, and AF9/ENL to form the multi-subunit complex termed the “super elongation complex” (SEC). AFF1/AFF4 are molecular scaffolds, ENL/AF9 interact with Pol II on chromatin, and ELL1/2 prevents the backtracking of RNA polymerase II [141,142,143]. Therefore, Tat is able to recruit SEC to the nascent viral transcripts causing the largest subunit of RNA polymerase II to be phosphorylated at Ser2 and Ser5 by CDK9 and the multi subunit TFIIH transcription factor, respectively. CDK9 also phosphorylates negative transcription elongation factors, like NELF, causing its release, and DSIF, turning it into a positive elongating factor, thus determining the transition from the initiation to the fully elongated stage of transcription [143,144,145], (Figure 1).

## 3. To “Shock” and to “Kill”

### 3.1. Latency Reversing Agents (LRAs) Promoting HIV-1 Transcription Initiation and Elongation in CD4^+^ T Cells 

Among different LRAs than can be used for therapeutic intervention, the PKC agonists appear to be the most efficient in driving the initiation of HIV-1 transcription and inducing escape from latency in J-Lat cell lines, latent primary CD4^+^ T cells, and in ex vivo T cells derived from aviremic patients [146]. Bryostatin-1, Prostratin, and Ingenol—all represent PKC agonists tested as LRAs. Nevertheless, the activation of multiple pathways using combinations of LRAs was most efficient as a reactivation protocol [71], while use of single LRA compounds resulted in poor reactivation. For example, Ingenol, together with the HDI Romidepsin, resulted in consistent HIV-1 reactivation in most subsets of latently infected CD4^+^ T cells from HAART-treated patients [147]. Synergistic reactivation was also obtained by the combination of Ingenol plus Romidepsin [147].

In other studies, Hexamethylenebisacetamide (HMBA), which causes the release of P-TEFb from HEXIM1 and the 7SK snRNPs [148], when combined with PKC agonists produced a synergistic re-activation, mediated by P-TEFb and NF-κB induction [149]. Similarly, combined treatment of the Ca^2+^ ionophore ionomycin with PKC activators resulted in a full transactivation on HIV-1 and nuclear translocation of RelA [118].

**Figure 2:**
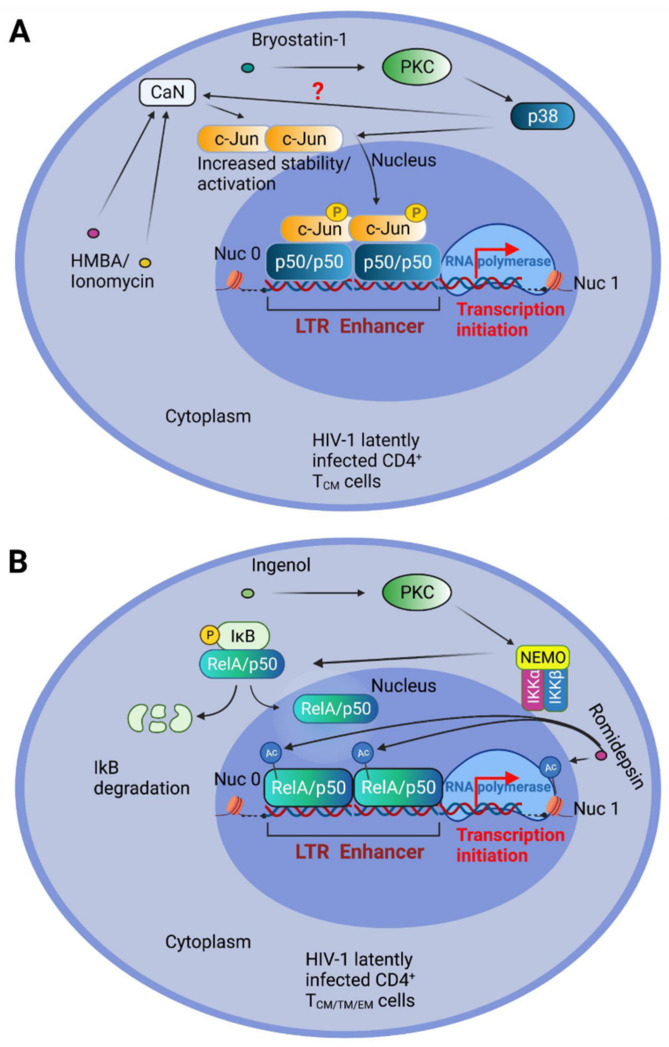
Schematic representation of the proposed molecular mechanisms acting in different subsets of CD4^+^ T central memory (_CM_) cells driving HIV-1 transcription initiation. (**A**) Suggested molecular mechanisms acting in CD4^+^ T central memory (_CM_) cells driving HIV-1 transcription initiation, following Bryostatin-1 plus Ionomycin or HMBA treatments. Bryostatin-1 drives protein kinase C (PKC) activation that, in turn, activates p38 kinase. Ionomycin and, to a lesser extent, Hexamethylenebisacetamide (HMBA), activate Calcineurin (CaN) phosphatase, that, together with p38 produce the accumulation of c-Jun transcription factor. Full c-Jun activation is obtained by its p38-mediated phosphorylation. Activated c-Jun binds the NF-κB inhibitory dimer p50/p50, bound to the long terminal repeat enhancer (LTR), determining initiation of HIV-1 transcription. The nucleosomes (Nuc 0) and Nuc1 -are also indicated. (**B**) Schematic representation of the latency reversing action of the combined treatments with Ingenol and Romidepsin, effective in almost all latently infected resting memory CD4^+^ T cell subtypes. The PKC agonist Ingenol activates PKC, which in turn stimulates the IκB Kinase (IKK) complex to phosphorylate IκB, determining its degradation. NF-κB is therefore free to accumulate in the nucleus and to bind the LTR enhancer. Full start of transcriptional activity is obtained by simultaneous treatment with the histone deacetylase inhibitor (HDI) Romidepsin, able to determine acetylation of nuc1 and of crucial NF-κB residues.

The NF-κB RelA/p50 heterodimer is crucial for the reactivation of the latent HIV-1 LTR in primary CD4^+^ T cells stimulated by PKC and Ca^2+^ pathway activators [118]. Nevertheless, prolonged NF-κB activation can lead to autoimmune, inflammatory, and malignant disorders. Using the stable expression of an NF-κB super-repressor (IκB-α 2NΔ4), our group has demonstrated that a synergistic reactivation from latency is achieved by the concomitant engagement of MAP kinase, and Ca^2+^/CaN, pathways in the J-Lat latency model using the PKC activator Bryostatin-1, together with the differentiating agent HMBA or the calcium ionophore Ionomycin [150]. We have also demonstrated that such combinations display the accumulation and activation of c-Jun. Indeed, CaN-mediated dephosphorylation of c-Jun at Ser-243 resulted in increased c-Jun protein stability, or nuclear accumulation when combined with phorbol 12-myristate 13-acetate (PMA) treatment [151,152]. Moreover, we demonstrated that c-Jun is crucial for latency reactivation by indirectly binding to the LTR enhancer κB sites in association with the p50 transcription factor [150] (Figure 2A). We also obtained comparable results using a primary CD4^+^ T central memory (T_CM_) cell latency model in the presence of acetylsalicylic acid (ASA), a potent inhibitor of the NF-κB kinase IKK-β. The combined treatment of Bryostatin-1 and the HDI Panobinostat effectively reversed latency in T_SCM_ cells [147]. On the other hand, Panobinostat, when tested in combination with Ingenol, had strong antagonistic effects on viral reactivation in CD4^+^ T cells [147]. These different outcomes, obtained with diverse PKC agonists, may be explained by a preferential IKK-NF-κB activation capacity of Ingenol (Figure 2B).

The so-called non-canonical NF-κB pathway is triggered following TNF receptor family members, like FN14, the lymphotoxin β receptor (LTβR), and CD40, and involves the activation of the NF-κB inducing kinase (NIK), which subsequently phosphorylates the NF-κB family member p100, causing its cleavage by the proteasome to generate the p52 subunit; p52, bound to the other NF-κB family member RelB, migrates to the nucleus to activate transcription [153,154]. The inhibitors of apoptosis cIAP E3 ubiquitine ligases can determine the degradation of NIK, and therefore are inhibitors of the NF-κB non-canonical pathway [154]. c-IAPs have been implicated as negative regulators of HIV-1 transcription, and therefore as latency promoters, while small molecules able to bind and antagonize c-IAPs, mimicking the endogenous c-IAP antagonist, second mitochondrial activator of caspases (SMAC), have been proposed as a novel class of LRAs [155,156,157] capable of reversing latency in animal models [158], and also promoters of the killing of latently infected cells [159].

IL-15 stimulation has been at the center of new studies, promoting its use or the exploitation of the IL-15 activated pathway, for either the shock, or the kill phase of the Shock and Kill approach. In this respect, IL-15 super agonist N-803 was used and was effective to determine latency reversal in HAART-treated macaques and humanized mice infected with simian immunodeficiency virus (SIV) and HIV-1, respectively, when combined with the depletion of CD8^+^ T lymphocytes [160], also showing the role of these cells in contrasting HIV-1 latency reversal in vitro [160]. Soluble recombinant human IL-15 (rhIL-15) has also been shown to stimulate natural killer (NK) cells to clear HIV-1-infected cells following latency reversal ex vivo, obtained by using the HDi Vorinostat [161].

**Figure 3:**
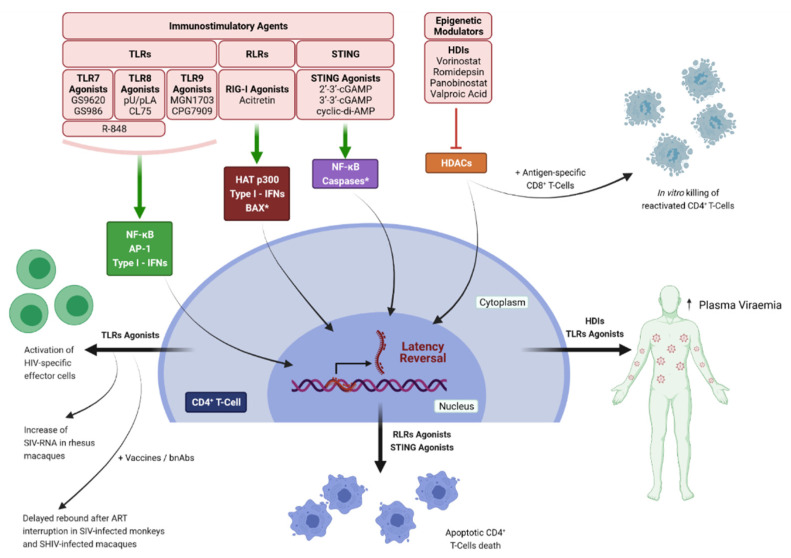
Schematic representation of LRAs mechanism of action. Histone Deacetylase Inhibitors (HDIs) induce histones hyperacetylation, which leads to a chromatin conformational change, activating transcription, thereby causing latency reversal; HDIs increase plasma viraemia in HIV-1 positive patients and, when administered together with HIV-specific CD8+ T-cells, induce selective killing of reactivated CD4+ T-Cells in vitro. TLRs agonists reactivate HIV-1 replication in a NF-κB, AP-1, and Type-I IFNs—dependent manner; TLRs stimulation also results in increased plasma viraemia in patients and in SIV-infected macaques, and triggers an HIV-specific adaptive response. Combination of TLRs agonists with therapeutic vaccines or broadly-neutralizing antibodies (bnAbs) protected SIV-infected monkeys and SHIV-infected macaques from viral rebound after ART cessation. RLRs agonist Acitretin stimulates latency reversal by activation of Histone Acetyl-Transferase p300 mediated by type-I IFN; STING agonists induce reactivation in a NF-κB dependent manner. Both RLRs and STING agonists induce selective apoptosis of reactivated cells. * Activated in apoptotic signaling.

Histone deacetylase inhibitors (HDIs) represent the most studied epigenetic LRA drugs and Romidepsin, Panobinostat, Vorinostat, and Valproic acid have been extensively studied in clinical trials. Despite initial promising results, these LRAs failed in clearing or eliminating the viral reservoir [162,163,164,165,166,167,168]. In vivo administration of Vorinostat increased the levels of cell-associated unspliced (CA US) HIV-RNA, but not plasma viral loads in HIV-positive patients [163,169,170,171]. In contrast, treatment with Panobinostat or Romidepsin enhanced both CA US HIV-RNA and plasma viremia [164,165]. However, none of these treatments resulted in a reduction in the viral reservoir, indicating that latency reversal alone is not sufficient to obtain viral clearance. Some studies suggest that a combined treatment could lead to the killing of HIV reactivated cells, at least in vitro; in fact, the addition of immune effector cells such as antigen-specific CD8+ T-cells, or the recruitment of NK cells induced by HIV-specific antibodies, resulted in the elimination of infected cells after reactivation [172,173]. Despite this encouraging results, no measurable reduction in the viral reservoir has been achieved in vivo [174,175] (Figure 3).

### 3.2. Innate Immunity Signaling Pathways Exploited for “Shock and Kill”

To obtain the elimination of reactivated cells, new classes of LRAs with the ability to modulate the innate immune response are currently being tested. These molecules have the potential to reactivate latent provirus and stimulate the immune system to target and kill infected cells. During acute infection, pattern recognition receptors (PRR), including the Toll-like receptors (TLRs), the cGAS–STING cytosolic DNA-sensing pathway, and the retinoic acid-inducible gene I (RIG-I) pathway, recognize pathogen-associated molecular patterns (PAMPs) and damage-associated molecular patterns (DAMPs), and subsequently induce apoptosis in virus-infected cells [176,177,178,179]. Different ex vivo and in vivo studies demonstrated that PRRs agonists reactivate latent HIV and simian immunodeficiency virus (SIV) and, at the same time, stimulate a specific anti-HIV CD8^+^-T and NK cell response [180,181,182,183,184]. 

Among the various PRRs, TLRs—mainly TLR 7, 8 and 9—have been extensively studied as LRAs. TLRs are expressed on different cells of the immune system including natural killer (NK) cells, macrophages, B cells, dendritic cells (DCs), and T cells, as well as epithelial and endothelial cells. TLR 7 and 8 senses single-stranded RNA, while TLR 9 senses unmethylated CpG-oligodeoxynucleotide-containing DNA on the endosomal membranes; this stimulation leads to the activation of several transcription factors, including NF-κB, AP-1, and interferon regulatory factors (IRFs) that induce the expression of inflammatory cytokines and type I interferons (IFNs) to protect the host from microbial infection. TLR7 agonists were reported to induce latency reversal in cells obtained from HIV-1 positive individuals and in SIV-infected rhesus macaques as well as a reduction in viral reservoir in the animals [181,182]. An increase in extracellular HIV-RNA levels in the ex vivo stimulation of PBMCs from HIV-1 infected patients undergoing ART was observed with the TLR 7 agonist GS-9620; this effect relied on type I IFN signaling activation in plasmacytoid dendritic cells and resulted in the activation of HIV-specific effector cells [182]. In an in vivo study on SIV-infected rhesus macaques on suppressive ART, it has been demonstrated that the TLR 7 agonists GS-986 and GS-9620 were able to induce the expression of SIV-RNA along with the activation of innate and adaptive immune response. This treatment also resulted in the reduction in SIV-DNA in ex vivo cell cultures. Furthermore, two out of nine animals did not show any viral rebound for more than 2 years after HAART interruption, and adoptive cell transfer from aviremic animals to naïve macaques did not result in de novo infection [181]. Unfortunately, these encouraging results were not reproduced in other studies involving non-human primates [185,186,187].

To improve the efficacy of TLR 7 agonists, different combinations with therapeutic vaccines and broadly neutralizing antibodies (bnAbs) have been tested. The combination of GS-9620 with the vaccine AD26/MVA led to innate and adaptive immune stimulation, a delay in viral rebound after ART cessation, and reduced viral DNA in lymph nodes and the peripheral blood of SIV-infected monkeys [187]. Similar results were obtained in rhesus macaques infected with Simian Human Chimeric Immunodeficiency virus (SHIV), receiving GS-986 together with two bnAbs, N6-LS and PGT121, where a modest delay in the viral rebound after ART interruption was observed [188]. However, in a double-blind, multicenter, placebo-controlled trial, no changes in plasma viral loads were observed in HIV-1 positive patients under ART treated with GS-9620, despite a consistent induction in IFN response and lymphocyte activation [189]. 

The ability of TLR 8 agonists to stimulate provirus transcriptional reactivation was assessed in resting CD4^+^ T cells isolated from the blood of nine virologically suppressed patients, in the presence or not of gamma-irradiated PBMCs and PHA [190]. The two synthetic ligands, pU/pLA and CL75, reversed latency in all patients and in three patients, respectively; in addition, these molecules were able to increase cytokine production and induce the upregulation of T cells’ surface activation markers [190]. Furthermore, the TLR7/8 agonist R-848 induced HIV-1 reactivation from latency in promonocytic cell lines [191], and in a subsequent work, it was demonstrated that the stimulation of TLR8 in monocyte-derived myeloid dendritic cells (MoMDCs) resulted in TNF-α—mediated—NF-κB—dependent reactivation of HIV, in an autocrine and paracrine manner [192]. TLR9 agonists can reactivate HIV from latently infected cells in vitro [193,194]. Among TLR9 agonists currently tested in vivo, MGN1703 was shown to increase plasma HIV-RNA levels in 6 out of 15 virologically suppressed patients on HAART [195]; this phenomenon was accompanied by the enhancement of immune responses, as indicated by the activation of plasmacytoid dendritic cells (pDCs), production of IFN-α2, upregulated transcription of IFN stimulated genes (ISGs) in CD4^+^ T cells, and activation of effectors cells [195]. However, despite a significant T-cell activation and an HIV-specific T-cell response, no changes in reservoir were observed in a following study [196]. A slight decrease in proviral DNA was observed in 31 HIV-positive patients treated with the pneumococcal vaccine, using the TLR9 agonist CPG7909 as an adjuvant [197]; this decrease correlated with the upregulation of HIV-specific CD8^+^ T cells, while no HIV-specific CD4^+^ T cells or HIV-specific antibodies were detected [197]. Collectively, TLRs agonists have shown promising activity as LRAs, especially when used in combination with bnAbs or as vaccine adjuvants; however, in vivo studies highlighted a lack of reproducibility in reducing the viral reservoir, suggesting that further improvements are needed.

In addition to TLRs, other PRRs have shown a potential as LRAs. The cytosolic sensors, RIG-I-like receptors (RLRs), and stimulator of interferon genes (STING) reactivate HIV from latency through p300 and NF-κB, respectively [198,199]. The RIG-I agonist Acitretin, a retinoic acid derivative, reactivated latent HIV and concomitantly induced apoptosis of cells actively transcribing HIV, in an IRF-3-dependent manner [198]. Moreover, a significant reduction in HIV-DNA levels was observed in an ex vivo stimulation with acitretin of CD4^+^ T cells obtained from 12 ART-suppressed HIV-positive patients, and the combination with the HDI Vorinostat further increase the magnitude of reactivation, the rate of apoptotic cell death, and the reduction in HIV-DNA [198]. However, a subsequent study failed to replicate these data in cell lines or in patient-derived samples [200]. A phase-I clinical trial to evaluate the effect of acitretin on immune cells and the expression of surface markers on CD4^+^ T cells has been planned (NCT03753867).

Another study demonstrated that STING agonists 2′-3′-cGAMP and cyclic-di-AMP induced SIV Gag RNA and reduced SIV Gag DNA in PBMCs from cynomolgus monkeys and demonstrated natural SIV control at 40 weeks post-infection [201]; furthermore, cyclic-di-AMP increased the frequency of SIV Gag-specific CD8^+^ T cells and stimulated reactivation in human PBMCs in vitro [201]. Our group also reported the ability of 2′-3′-cGAMP to reactivate HIV in an NF-κB dependent manner, and to induce the selective apoptosis of latently infected cells in vitro [199]. These effects were potentiated when cGAMP was used in combination with the HDI Resminostat. In addition, the cGAMP stimulation of a primary model of latency based on CD4^+^ T_CM_ cells resulted in the selective death of HIV-1 harboring cells even in the absence of reactivation. cGAMP was also able to induce high levels of cell-associated HIV RNA in PBMCs and CD4^+^ T cells obtained from HIV-1 positive patients on HAART with undetectable levels of viremia [199]. Recently, 2′3′-cGAMP and 3′3′-cGAMP were found to be potent adjuvants for the induction of antigen-specific CD8^+^ T cells in HIV-1 Gag p24 vaccinated mice; this response was dependent on the induction of type I IFNs [202], (Figure 3).

## 4. To “Block” and to “Lock”

In an apparent contrast to what the Shock and Kill approach tries to achieve, some of the research focusing on a functional cure aims to definitively silence the HIV-1 integrated provirus. By targeting viral and cellular factors, playing an important role in HIV transcription and silencing, and the pathways they are involved in, it could be possible to implement the so called “Block-and-Lock” approaches by using small molecules called latency-promoting, or latency inducing, agents (LPAs/LIAs). Among these, one of the most advanced employs didehydro-cortistatin A (dCA), a Tat inhibitor that affects the function of the viral transactivator crucial in HIV-1 transcriptional elongation [203]. Moreover, the suppression of HIV-1 LTR transcription by the dCA inhibition of Tat function (“block”) has been associated with a progressive accumulation of chromatin repressive features (“lock”), specifically occurring at the HIV-1 LTR and especially involving Nuc1 [204], thus reinforcing the hypothesis that a permanent silencing of HIV-1 transcription could be achieved by a prolonged treatment with LPAs. In this respect, Triptolide, which stimulates the proteasomal degradation of Tat, could be useful in the context of such an approach [205], and a clinical trial aimed at studying the impact of Triptolide on HIV-1 reservoir in acute HIV-1 infection is on-going (https://clinicaltrials.gov/ct2/show/NCT02219672, accessed on 10 November 2021).

Inhibitors of the interaction between HIV integrase (IN) and the cellular chromatin-tethering factor LEDGF/p75 (LEDGINs) were suggested to implement a block-and-lock approach. As a consequence of treatment with LEDGINs in cell culture, the integrating viruses are repositioned out of active genes. Retargeted proviruses are predominantly in a latent state, and are refractory to reactivation by LRAs [206]. Such a class of small molecules could be employed during chronic infection to reduce the pool of reactivatable HIV-1 genomes in case residual viral replication occurs, or after treatment interruption if viral rebound takes place, coupled with HAART [207].

Curaxin CBL0100-mediated inhibition of the facilitates chromatin transcription complex (FACT), a newly identified regulator of HIV-1 transcription [208], acting as a histone chaperone capable of destabilizing the nucleosome structure and, as a consequence, promoting RNA pol II-driven transcription, represents an additional option to suppress HIV transcription [209].

An alternative Block and Lock approach involves the use of siRNA or shRNA to prevent the binding of specific transcription factors to the 5 ‘LTR, with the purpose of maintaining heterochromatin in a repressive state at the HIV-1 promoter. This approach is known as RNA-Induced Epigenetic Silencing of HIV transcription [210,211].

The Heat shock protein 90 (Hsp90) plays a role in HIV-1 reactivation due to its ability to stimulate NF-κB-dependent latency reversal mediated by PKC-activation [212]. Therefore, HSP90 inhibitors are being investigated to suppress viral transcription. In this respect, the administration of specific Hsp90 inhibitors in clinical development, like tanespimycin (17-(allylamino)-17-demethoxygeldanamycin) and AUY922, prevented viral rebound in HIV-infected humanized NOD scid IL-2Rγ^−/−^bone marrow-liver-thymus mice up to 11 weeks after treatment interruption [213].

The involvement of the Jak-STAT pathways in latency/latency reversal has also been hypothesized, since two FDA approved Jak inhibitors, Ruxolitinib and Tofacitinib, thanks to their anti-inflammatory effects and regulatory action on HIV transcription, are able to prevent the reactivation of HIV-1 replication by LRAs in primary CD4^+^ T cells [214]. Moreover, Ruxolitinib and Tofacitinib target the signal transduction pathways downstream of γ-C cytokine (IL-2, IL-7 and IL-15) receptors engagement, thus having an impact on the HIV reservoir in all memory CD4^+^T cell subsets in vitro and ex vivo [215]. A clinical trial is on-going to evaluate the safety and tolerability of Ruxolitinib in antiretroviral-treated HIV-infected adults (https://clinicaltrials.gov/ct2/show/NCT02475655, accessed on 10 November 2021).

It has been experimentally demonstrated in in vitro and ex vivo cell models that ZL0580, a novel and more specific inhibitor of BRD4, induced HIV-1 suppression. Moreover, in cells from aviremic patients under HAART, ZL0580 determines a delay in the rebound of viral replication after the drug combination was removed from cells [216]. These results are apparently in contrast with those obtained with the BET/BRD4 pan-inhibitor JQ1, which was instead shown to promote HIV-1 escape from latency [217]. Nevertheless, BRD4 is versatile in regulating target gene expression, and could exert positive or negative effects on HIV proviral transcription depending on its partner proteins [218]. Moreover, many lines of evidence suggest a crucial role for BRD4 in the early phase of HIV-1 transcription when Tat is not yet available [121].

Dual mTORC1 and mTORC2 inhibitors, like Torin1 and pp242, have been shown to be effective in suppressing HIV reactivation from latency by decreasing CDK9 phosphorylation induced by TCR co-stimulation in CD4^+^ T cells and possibly resulting in NF-κB blockade, and more potently inhibit HIV-1 compared to the more specific mTORC1 inhibitor Rapamycin [219]. Therefore, the use of such inhibitors, together with other latency promoting agents, could be useful to implement a combined Block and Lock strategy [220].

Finally, several kinase inhibitors are under investigation to evaluate their potential use for the “Block and Lock”, and have shown some effectiveness in preventing reactivation by LRAs [221].

## 5. Summary & Perspectives

Many hurdles remain on the road to an HIV-1 cure. The presence of viral sanctuaries in specific tissues, characterized by low penetration of HAART and immune-privileged sites, may be the source of potential residual viral replication and persistence [71].

Another challenge to latency reversal strategies is the heterogeneous nature of cell subtypes harboring a replication competent integrated HIV-1 provirus. The Shock and Kill approach to an HIV cure is further complicated by the observation that only a fraction of HIV-1 latently infected cells harbor replication competent proviruses [222], as well as the deep latency state of non-defective HIV-1 proviruses [70]. Moreover, different LRAs and combinations appear to be required for the in vivo reactivation of HIV-1 in different latent cell types [147].

The efficient reactivation of HIV-1 transcription in cell reservoirs following specific LRA treatments does not necessarily result in the production of viral proteins [147] or cell death, due to a reduction in HIV-1 specific cytotoxic T lymphocyte (CTL) responses [222]. The overexpression of the prosurvival factor B cell lymphoma 2 (BCL-2) in CD4^+^ T cells after co-culture with the corresponding HIV-CTL argues that BCL-2 may be a potential therapeutic target for the shock/kick and kill approach [223].

The importance of the anti-apoptotic Bcl-2 protein in promoting the prolonged survival of cells replicating HIV-1 is demonstrated by the use of Venetoclax, a Bcl-2 antagonist, that leads to HIV-1 productively infected primary T-cells selective killing [224], and also reduces the frequency of latently-infected T-cells from HAART patients when combined with anti-CD3/CD28 stimulation [225]. Furthermore, the role of the pro-survival pathway engaged by the activation of the Pi3K-AKT kinase axis for the replicating HIV-1 is evidenced following the activation of this pathway by the HIV-1 Nef and Tat proteins, thus preventing the premature apoptosis of HIV-1 infected and replicating T-cells [226]. AKT inhibitors work in HIV-1 infected macrophages, and their function in T cells has to be confirmed [226].

The identification of markers in latently infected cells would allow the specific targeting of these cells by LRAs stimulation. However, to date, the identification of T cell specific markers of latency—such as CD32a—is still debated [71].

Despite the enormous diversity of HIV-1 subtypes, most studies have been performed using subtype B virus sequences. In this regard, the virus subtype may have an impact on both the establishment of and reactivation from latency [227].

A promising strategy derives from the use of immunostimulatory agents such as LRAs to induce an adaptive response against latently infected cells. In animal models, TLR agonists induced the expression of viral RNA, and activation of both innate and adaptive immune response [181]. These effects were further enhanced when combining the agonists with therapeutic vaccines or bnAbs [187,188]. Recently, RLRs and STING agonists have emerged as a promising new class of LRAs, with the capacity to induce the latent provirus and stimulate the selective apoptosis of reactivated cells [199]. Despite a robust immune activation elicited by these compounds, it remains a challenge to extend the in vitro results to HIV-1 positive patients. Further studies are needed to identify and subvert the mechanisms that protect latently infected cells from reactivation and/or elimination by immune system in vivo.

One of the problems related to the shock phase of the Shock and Kill approach is also related to the activation, by different LRAs, of signal-transduction pathways associated with increased cell survival, such as in the case of the canonical NF-κB activation pathway, thus posing an additional challenge to the following “kill” phase. The use of SMAC mimetics may be one of the possible solutions, exerting a negative regulatory effect on the canonical pathway, while activating the non-canonical one and promoting apoptosis [154]. A down side of the non-canonical NF-κB pathway, however, is that it also modulates the effector function of differentiated T cells, potentially promoting TH17 cell-mediated neuroinflammation [228].

The Block and Lock approach is moving forward, offering an alternative to the many hurdles the “Shock and Kill” is facing. The idea of blocking LTR-mediated transcription with LPAs and to induce, as a consequence, a long-lasting repressive chromatin signature is intriguing and reminiscent of human endogenous retroviruses (HERVs) that are apparently transcriptionally silent [229]. HERVs can be stimulated by environmental stress and pathogenic infections to reactivate, thus producing envelope (ENV) proteins that have been associated with neuropsychological diseases [230]. Moreover, the HIV-1 reservoir present in the central nervous system (CNS) may be pushed by LRAs to produce viral proteins, like Tat, able to be released from infected cells and to exert a detrimental effect on neurons and in general on the CNS, arguing against the “Shock and Kill”. On the other hand, the fact that HERV lock-down may not be permanent suggests that a long lasting “lock” of the latent HIV-1 may not be easily achievable. The “Block and Lock” may work better with an HIV-1 genome integrated in transcriptionally silent chromatin regions, but poorly with proviruses close to actively transcribed genes and, on top of that, will not reduce the size of the reservoir.

**Figure 4:**
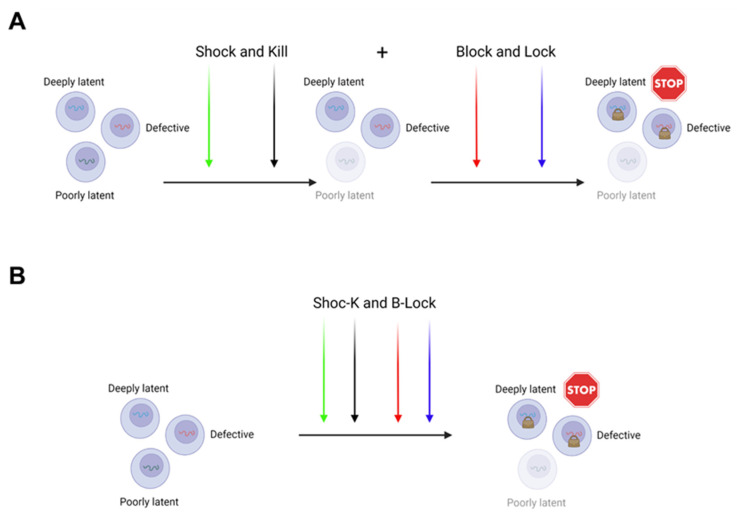
Schematic representation of two different possibilities to combine the “Shock and Kill” and the “Block and Lock” approaches to reach a functional cure. (**A**) The sequential application of the “Shock and Kill” followed by the “Block and lock” could lead to an initial reduction/elimination of poorly latent reservoirs, easy to be reactivated by LRAs, followed by the blocking and locking of potential residual replication by LPAs. (**B**) “Shoc-K and B-Lock”. All-in-one cocktail of LRAs + LPAs could result in a tailored reactivation and killing of poorly latent reservoir and, at the same time, the long term locking of residual deeply latent proviruses.

Therefore, the “Shock and Kill” and the “Block and Lock” may not only be alternative to each other, but in fact, can be merged and proposed together to achieve a functional cure (Figure 4).

Two ways can be envisioned to do so. In the first, a sequential application of both approaches (“Shock and Kill” first, followed by “Block and Lock”) could eliminate those cells where the virus is easy to be targeted for reactivation by LRAs, and subsequently a deeper silencing could be achieved by LPAs in deep seated latent residual proviruses (Figure 4A). The second possibility is to realize the “Shoc-K(Kill) and B(Block)-Lock” by using a unique all-in-one cocktail of compounds possessing tailored activities to promote both latency reversal and latency induction, resulting in a reduced and deeper silenced reservoir (Figure 4B). In this respect, we have demonstrated that the combination of activators of MAPKs and Calcineurin, like Bryostatin-1 and HMBA, used with acetylsalicylic acid (ASA) at a high dosage, the latter capable to strongly inhibit canonical NF-κB activation, is able to determine a synergistic reactivation of HIV-1 transcription from the JLat 10.6 cell line and a strong reactivation of transcription in a CD4+ T_CM_ cell primary latency model [150]. Considering the role of the canonical NF-κB pathway activation in cell survival, its inhibition by Aspirin from one side could inhibit the CD8^+^ T cell-mediated clearance of cells that reactivate viral replication, due to the Aspirin inhibition of T-cell activation and proliferation; on the other hand, it could promote apoptosis in those cells, where activated c-Jun/p50 complex is enough to drive efficient transcription initiation, by binding to the LTR enhancer, also avoiding a potentially toxic effect related to a separated and sequential application of both approaches.

While intriguing, this theoretical approach awaits experimental testing and an experimental setting able to test the efficacy of each step of the “Shoc-K and B-Lock”.

## Data Availability

The datasets generated during and/or analysed during the current study are available from the corresponding author on reasonable request.
